# Beyond phospholipid remodelling: biosynthetic roles of lysophospholipid acyltransferases

**DOI:** 10.1042/BST20250125

**Published:** 2026-11-12

**Authors:** Kasparas Petkevicius

**Affiliations:** MRC Mitochondrial Biology Unit, University of Cambridge, Cambridge Biomedical Campus, Cambridge, U.K.

**Keywords:** acyl chain remodelling, lipidomics, lysophospholipid acyltransferases, membrane composition, phospholipid remodelling, phospholipids

## Abstract

Phospholipid remodelling is commonly used as an umbrella term for enzymatic changes in phospholipid fatty acyl chain composition after initial synthesis. This process is typically mediated by the Lands cycle, in which phospholipase-generated lysophospholipid intermediates are reacylated by lysophospholipid acyltransferases (LPLATs). However, not all lysophospholipid acylation reactions serve the same biological purpose. Some LPLAT activities remodel pre-existing phospholipid classes, tuning membrane properties by altering acyl chain composition. Others act as committed biosynthetic steps, generating defined phospholipid products whose acyl chains are integral to their identity and function. This distinction is essential for interpreting both enzyme function and lipidomics data, because individual phospholipid species may represent either molecular variants within a broader class or products with specific biological roles. In this review, I argue that grouping all lysophospholipid acylation reactions under the single label of phospholipid remodelling can obscure important biological differences. Separating remodelling from biosynthetic functions of mammalian LPLATs provides a clearer framework for understanding phospholipid metabolism, membrane organisation and mechanisms linking phospholipid metabolism to human disease.

## Introduction

In the late 1950s, William E.M. Lands carried out a series of radiotracer and biochemical studies that transformed how phospholipid metabolism was understood [1–3]. At the time, phospholipid metabolism was framed primarily through *de novo* assembly pathways, particularly the Kennedy pathway [4]. Lands’ work shifted this view by showing that phospholipid fatty acyl groups are dynamically removed and re-incorporated through a cyclic process involving lysophospholipid intermediates [1–3]. These studies established the basis for what later became known as the Lands cycle: a pathway in which phospholipase A-mediated deacylation and LPLAT-mediated reacylation alter phospholipid acyl chain composition independently of *de novo* phospholipid synthesis [5]. In this review, phospholipid remodelling refers to such changes in acyl chain composition without alteration of headgroup identity. Specifically, I focus on the LPLAT-mediated reacylation arm of the mammalian Lands cycle and on how individual LPLAT reactions should be interpreted biologically.

In the decades following Lands’ original studies, lysophospholipid reacylation was characterised in increasing biochemical detail, but the enzymes responsible for these reactions have only been molecularly identified over the last two decades. These enzymes, now broadly termed lysophospholipid acyltransferases (LPLATs), generate phospholipids by transferring a fatty acyl chain to a free hydroxyl group on the glycerol moiety of a lysophospholipid acceptor [6]. In most cases, the acyl donor is an acyl-coenzyme A (acyl-CoA) thioester, with coenzyme A acting as a soluble carrier that presents defined fatty acyl groups, such as 16:0, 18:1, 20:4 and 22:6, for phospholipid assembly [6]. Cells can also reacylate lysophospholipids through acyl-CoA-independent transacylation, in which the acyl chain is transferred directly from one lipid to another. Thus, although lysophospholipid reacylation is a common chemical outcome, it can be achieved by mechanistically distinct enzymes.

In the acyl-CoA-dependent branch, LPLATs are distributed across several conserved protein families with different catalytic chemistries and structural architectures, including the AGPAT/LPLAT, MBOAT/LPLAT and TRAM–Lag1–CLN8–domain-containing (TLCD) families [6,7]. However, substrate selectivity is determined primarily at the level of individual enzymes rather than by phylogenetic grouping. Even closely related LPLATs can differ markedly in lysophospholipid headgroup preference, *sn*-position selectivity and acyl donor usage [6,7]. As a result, different LPLATs produce distinct phospholipid molecular species, allowing cells to build phospholipid pools with a wide range of acyl chain compositions. The nomenclature, biochemical diversity and acyl preferences of mammalian LPLATs have been reviewed extensively elsewhere [6].

What remains less well defined is how these enzymatic reactions should be interpreted biologically. This question has become increasingly important as modern lipidomics now provides detailed species-level information across many biological settings, while the functional meaning of these changes often remains difficult to assign. Here, I propose that LPLAT activity can be understood through two conceptually distinct modes. In one mode, lysophospholipid acylation is a committed biosynthetic step that generates a defined phospholipid end product, with its characteristic acyl composition forming an integral part of its biological identity and function. In the other, cycles of deacylation and reacylation operate as a homeostatic remodelling mechanism, reshaping the acyl chain landscape of pre-existing membranes to tune their biophysical properties and align membrane composition with cellular state. Distinguishing between these modes is therefore essential for understanding the biological functions of individual LPLATs and for interpreting the significance of the phospholipid species they produce.

At the outset, it is important to emphasise that these two modes are not mutually exclusive. LPLAT functions are context-dependent and often lie along a continuum: the same enzyme may contribute predominantly to remodelling in one cell type or physiological state, yet support the biosynthesis of a defined phospholipid in another, depending on factors such as substrate availability, subcellular localisation, competing pathways and metabolic demand. The distinction adopted in this review should therefore be viewed as a conceptual lens rather than a rigid classification. Here, I use it to examine when lysophospholipid acylation serves phospholipid biosynthesis, when it serves phospholipid remodelling, and how separating these roles helps clarify LPLAT function, phospholipid function and the principles governing membrane acyl chain organisation.

## Synthesis versus remodelling

Because biosynthesis and remodelling can be defined either by the position of a reaction within a pathway or by its biological outcome, I use these terms here in an operational, outcome-based sense. An LPLAT reaction is considered biosynthetic when it constitutes an obligatory, non-redundant step in the production or maturation of a phospholipid class. This can include reacylation reactions that occur downstream of *de novo* synthesis, when they are required to generate the mature, physiologically functional phospholipid class. The underlying biosynthetic route need not be chemically simple and may include headgroup exchange as well as cycles of deacylation and reacylation [8]. Blocking such a pathway may also cause accumulation of the upstream lysophospholipid intermediate that serves as the substrate for the disrupted reaction. Thus, a decrease in phospholipid class abundance, often accompanied by an increase in the corresponding lysophospholipid intermediate, provides the expected experimental signature of an LPLAT acting in a biosynthetic mode (Figure 1 and Table 1).

**Figure 1:**
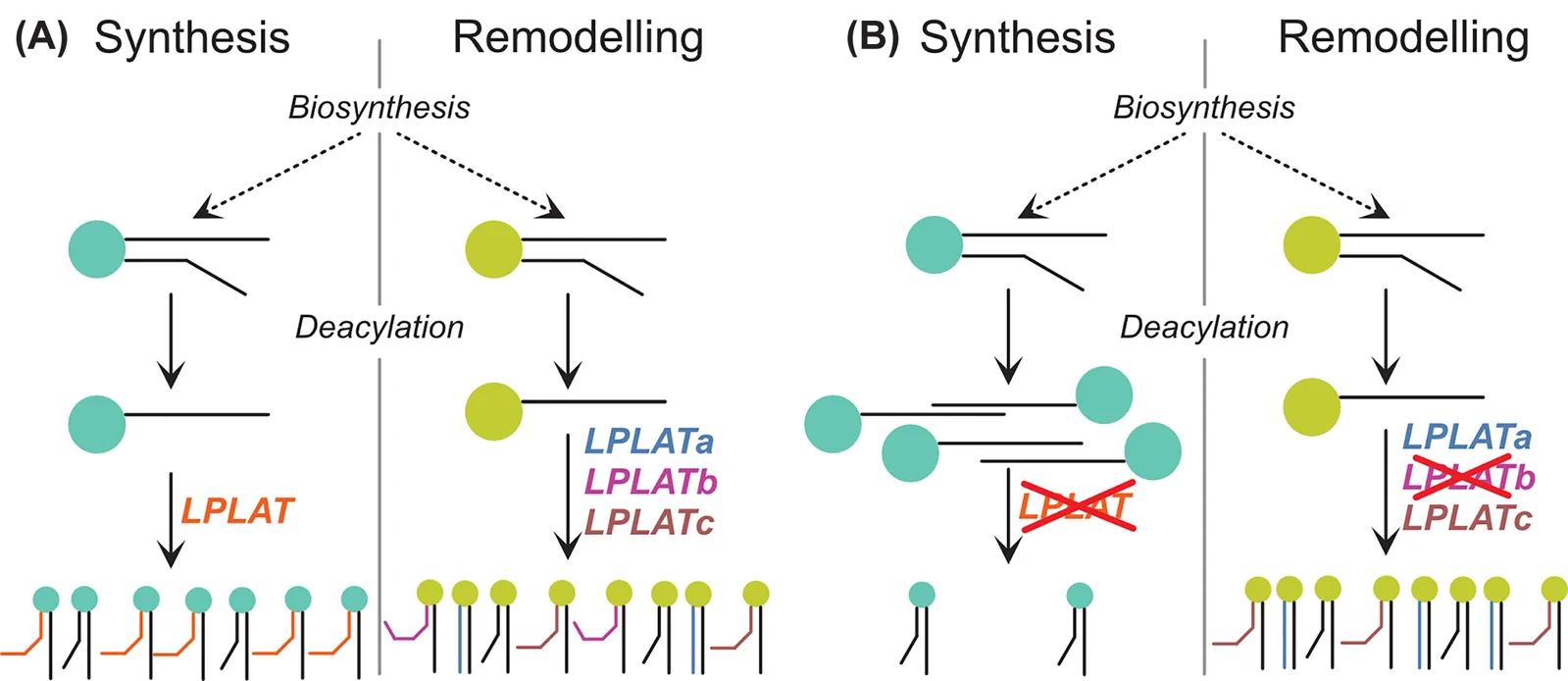
Schematic comparison of LPLAT function in phospholipid synthesis and remodelling Precursor synthesis and deacylation are common to both modes; the modes differ in how the resulting lysophospholipid is reacylated. (**A**) In biosynthetic pathways, a single LPLAT generates a dominant phospholipid pool with a defined acyl chain composition. By contrast, in phospholipid remodelling, multiple LPLATs act on the same lysophospholipid substrate to diversify and adjust the acyl composition of an existing phospholipid class according to cellular needs. (**B**) Loss of an LPLAT functioning in biosynthesis depletes the corresponding phospholipid product and leads to accumulation of the upstream lysophospholipid intermediate. By contrast, loss of a single remodelling LPLAT reduces molecular species diversity within that phospholipid class, while total class abundance is preserved through compensatory action of other LPLATs.

**Table 1:** Indicators of whether an LPLAT is acting in synthesis or remodelling

Question	Interpretation
Does enzyme loss reduce total abundance of the relevant phospholipid class?	A decrease in class abundance is consistent with a biosynthetic role. Preserved class abundance with altered species composition is consistent with remodelling.
Does the lysophospholipid substrate accumulate?	Substrate accumulation suggests a blocked step in a pathway. In remodelling, lysophospholipid levels are typically buffered by competing LPLATs, while acyl-CoAs no longer used by the missing enzyme can be redirected into other pathways, such as DGAT-mediated triacylglycerol synthesis.
Can other LPLATs compensate?	Compensation by enzymes acting on the same lysophospholipid substrate is typical of remodelling. Lack of compensation suggests that the enzyme occupies a committed biosynthetic position.
Is the product a defined lipid endpoint or part of a broad species pool?	Production of a stereotyped lipid species, such as PI 38:4 or tetralinoleoyl cardiolipin, is more consistent with biosynthesis. Diversification of PC or PE species is more consistent with remodelling.
Where does the enzyme act, and what substrates can it access?	Localisation, local acyl-CoA availability, lysophospholipid supply and cell state can all influence whether the reaction behaves primarily as synthesis or remodelling.

Distinguishing synthesis from remodelling requires understanding how an LPLAT contributes to lipid production and turnover. These questions bring together evidence from pathway position, substrate use and the consequences of enzyme loss. Changes in lipid abundance and acyl chain composition provide complementary clues, while stable isotope tracing can reveal whether the enzyme supports net synthesis, recycling of existing lipids, or both. Changes in lipid abundance should be interpreted alongside turnover and compensatory pathways, which can either amplify or mask the effects of enzyme loss.

By contrast, an LPLAT reaction is considered remodelling when its predominant role is to alter the acyl chain composition of a pre-existing phospholipid class rather than contribute to its net production. Consequently, loss of a remodelling enzyme leaves the total abundance of that phospholipid class largely intact while narrowing or shifting its molecular species profile (Figure 1). The distinction therefore depends on what the reaction achieves biologically.

This difference follows from how the pathway is organised. In phospholipid remodelling, the key step is often the generation of the lysophospholipid intermediate rather than its reacylation by one specific enzyme. Once that intermediate is produced, it can be reacylated by multiple LPLATs with overlapping substrate preferences (Figure 1). The final molecular species profile within that phospholipid class therefore reflects competition among different LPLATs, shaped by their expression, localisation and acyl donor selectivity. Loss of one remodelling LPLAT does not abolish production of that phospholipid class, but instead redirects flux towards other enzymes acting on the same lysophospholipid substrate, changing acyl chain composition rather than total class abundance (Figure 1). The distinction between the two modes therefore does not rest on how selective an individual enzyme is. A highly selective LPLAT can still act in remodelling if other enzymes can acylate the same lysophospholipid substrate, whereas an enzyme with broad acyl selectivity can occupy a biosynthetic position if alternative routes cannot adequately sustain production of the relevant lipid.

## The importance of distinguishing synthesis from remodelling

Whether an LPLAT functions in phospholipid synthesis or remodelling has major implications for the biological questions asked about that enzyme, the experimental strategies used to study it, and the interpretation of both its phospholipid product and lysophospholipid substrate.

When an LPLAT acts in a biosynthetic pathway, its reaction contributes directly to net production of a phospholipid class. This implies that the acyl chain composition generated by that LPLAT is likely to be an integral part of the function of the final phospholipid, rather than a context-dependent modification layered onto a pre-existing phospholipid pool. In this setting, a central biological question is why that particular molecular species is produced: what features of its acyl composition have been selected for, and what do they reveal about the function of the phospholipid and the pathway that makes it?

Studying biosynthetic LPLATs can therefore provide insight not only into pathway architecture, but also into the structural basis of phospholipid function. Loss of such an enzyme is expected both to reduce the relevant phospholipid class and to cause accumulation of the upstream lysophospholipid intermediate. The biological consequences of lysophospholipid substrate accumulation must therefore be considered alongside those of product depletion when interpreting the loss-of-function phenotype.

By contrast, when an LPLAT functions in phospholipid remodelling, its role is not to establish net synthesis of a phospholipid class, but to tune the molecular composition of an existing pool in ways that match cellular needs. In this context, reacylation is often adaptive: it optimises membrane properties for a particular cellular state, environment or function. Classic examples come from ectothermic organisms, which alter membrane acyl composition to maintain membrane fluidity across changing environmental temperatures [9–11]. More broadly, changes in acyl composition can influence membrane curvature, packing and flexibility, thereby helping membranes support processes such as organelle shape change, vesicle budding and trafficking [12–14]. The abundance of polyunsaturated phospholipids is also highly relevant in oxidative stress, where it can increase susceptibility to ferroptosis [15,16].

The consequences of losing a remodelling LPLAT may therefore be modest under basal conditions and become most apparent only when cells are challenged in a setting where that particular membrane composition becomes important. An enzyme may appear dispensable in one cell type or growth condition, yet become important when the relevant substrate pool, membrane demand or stress state changes.

## Examples of LPLATs in phospholipid remodelling

To illustrate this distinction, it is useful to begin with examples in which LPLATs function in clear phospholipid remodelling.

### Incorporation of PUFAs into the *sn*-2 position: LPCAT3/LPLAT12 and LPLAT3

LPCAT3, also termed LPLAT12 in the proposed unified nomenclature, is one of the best-characterised LPLATs [17,18]. It preferentially uses polyunsaturated fatty acyl (PUFA)-CoAs, particularly 18:2-, 20:4- and 22:6-CoA, to acylate lysophosphatidylcholine (LPC) and lysophosphatidylethanolamine (LPE) at the *sn*-2 position [17,19]. In multiple tissues and cell types, LPCAT3 deficiency markedly reduces PUFA content in phosphatidylcholine (PC) and phosphatidylethanolamine (PE) [20–24]. However, total PC and PE abundance is preserved, with PUFA-containing species replaced by species containing monounsaturated fatty acyl (MUFA) tails.

This pattern is characteristic of phospholipid remodelling. LPCAT3 does not determine whether PC and PE are produced, but rather which molecular species make up these phospholipid classes. LPCAT3-mediated phospholipid remodelling has important functional consequences. In enterocytes, PUFA-containing phospholipids help generate membrane properties required for triglyceride handling and lipoprotein assembly, and intestine-specific LPCAT3 deficiency impairs chylomicron formation and postprandial lipid absorption [21,22]. In hepatocytes, LPCAT3-dependent remodelling affects ER membrane composition, lipogenesis and lipoprotein secretion [20,24]. LPCAT3, therefore, provides a clear example of an LPLAT that remodels major phospholipid classes to match membrane function to physiological demand.

PUFA-containing phospholipids are not generated solely by Lands cycle reacylation. LPLAT3, also known as AGPAT3 or LPAAT3, acylates lysophosphatidic acid preferentially with 22:6-CoA, introducing DHA at the *sn*-2 position during *de novo* glycerophospholipid synthesis [25–28]. Loss of LPLAT3 markedly depletes DHA-containing phospholipids in tissues such as testis, retina and liver. This causes male infertility, abnormal photoreceptor disc morphology with impaired vision, and altered hepatic PUFA metabolism [25,26,28]. LPLAT3, therefore, acts upstream of the Lands cycle. Yet in functional terms it resembles LPCAT3: it determines which molecular species make up major phospholipid classes rather than whether these classes are produced.

### Remodelling at the *sn*-1 position: TLCD1 and LPEAT2/LPLAT10

Although phospholipid remodelling is most often discussed in relation to the *sn*-2 position, it also occurs at the *sn*-1 position. This is important because acyl chains at the *sn*-1 position, which are typically saturated fatty acids (SFAs) or MUFAs, make major contributions to bilayer packing and membrane order [13,29]. The relative abundance of palmitate (16:0), stearate (18:0), palmitoleate (16:1) and oleate (18:1) at the *sn*-1 position of major membrane phospholipids is therefore not simply a passive consequence of *de novo* synthesis, but is itself regulated through remodelling.

We recently identified TLCD1 as a previously unrecognised LPLAT that incorporates MUFAs into the *sn*-1 position of PE [7,30]. Loss of TLCD1 leaves total phospholipid class abundance unchanged, but shifts the composition at the *sn*-1 position from MUFAs towards SFAs [7,30], consistent with compensatory reacylation by enzymes using saturated acyl-CoAs, such as LPGAT1/LPLAT7 [31]. In the same work, we identified the related yeast protein, which we named ALE2, and showed that it performs an analogous role in controlling MUFA incorporation into PE [7]. Notably, neither TLCD1-deficient mice nor ALE2-deficient yeast show overt phenotypes under basal conditions.

LPEAT2, or LPLAT10, has likewise been implicated in MUFA incorporation into the *sn*-1 position of major phospholipids, particularly PC [32]. However, the phenotypes reported so far are relatively subtle: LPEAT2-deficient mice show only limited changes in selected MUFA-containing PC species in the brain and no overt abnormalities under basal conditions [32]. LPEAT2/LPLAT10 has also been shown to generate PC containing the very-long-chain saturated fatty acid C26:0, the precursor of the C26:0-LPC used as a diagnostic marker for X-linked adrenoleukodystrophy [33]. LPLAT6, also known as LCLAT1 or LYCAT, similarly incorporates stearate into the *sn*-1 position of PI, and LPLAT6-deficient mice show altered PI acyl composition but no overt phenotype [34].

Together, these examples are consistent with a remodelling role. Total phospholipid class abundance is preserved, but the balance between saturated and monounsaturated acyl chains at the *sn*-1 position is altered. A key outstanding question is why cells regulate this balance, and under which physiological conditions these differences become functionally important?

## Examples of LPLATs in phospholipid synthesis

By contrast, some LPLATs operate in pathways in which lysophospholipid acylation behaves as a committed biosynthetic step, determining both the acyl composition and the production of the relevant phospholipid endpoint.

### MBOAT7/LPLAT11 and PI 38:4

MBOAT7, also termed LPLAT11, is a lysophosphatidylinositol (LPI) acyltransferase of considerable biomedical interest. Biallelic loss-of-function mutations in MBOAT7 cause a neurodevelopmental disorder characterised by intellectual disability, developmental delay and seizures [35–37], whereas common MBOAT7 variants are strongly associated with metabolic dysfunction-associated steatotic liver disease [38,39]. Mechanistically, MBOAT7 catalyses acylation of LPI with arachidonoyl (20:4)-CoA, generating phosphatidylinositol (PI) species enriched in 20:4, in particular 18:0-20:4 PI, or PI 38:4 [40]. PI then serves as the precursor for headgroup-phosphorylated phosphoinositides that regulate membrane trafficking and signal transduction.

Although this is chemically a reacylation reaction, several features suggest that MBOAT7 is better viewed as functioning in biosynthesis of the canonical PI pool rather than in PI remodelling. Unlike PC or PE, PI and its phosphorylated derivatives show remarkably restricted acyl composition in most mammalian cells and tissues, with 18:0-20:4 species making up the great majority of the class [41]. In addition, MBOAT7 deficiency reduces total PI abundance and causes accumulation of LPI, indicating that other LPI acyltransferases cannot compensate sufficiently to maintain normal PI levels [42–45]. Thus, cells do not appear to use MBOAT7 simply to diversify PI species; rather, they appear to use it to generate the dominant PI 38:4 pool.

Framed in this way, the central question becomes why phosphoinositide biology is built so strongly around a conserved 18:0-20:4 scaffold. Why has this particular molecular species been selected so strongly over others? One possibility is that its acyl composition provides the optimal balance for diffusion within membranes, recognition by phosphoinositide-binding proteins, or access to the enzymes that phosphorylate, dephosphorylate and hydrolyse PI. These possibilities remain largely unexplored.

### Tafazzin and tetralinoleoyl cardiolipin

Tafazzin is another LPLAT with major pathophysiological relevance. Loss-of-function mutations in *TAZ*, which encodes tafazzin, cause Barth syndrome, a mitochondrial disorder characterised by cardiomyopathy, growth delay and mitochondrial dysfunction [46,47]. Tafazzin acts on cardiolipin (CL), a signature mitochondrial phospholipid enriched in the inner mitochondrial membrane, where it supports membrane curvature, respiratory chain organisation and mitochondrial bioenergetics [48]. Unlike acyl-CoA-dependent LPLATs, tafazzin transfers acyl chains between phospholipids and monolysocardiolipin (MLCL). Through repeated cycles of deacylation and transacylation, this reaction enriches CL with linoleate (18:2), generating tetralinoleoyl CL as the dominant mature species in many mammalian tissues [49–51]. Importantly, this enrichment does not appear to reflect intrinsic specificity of tafazzin for 18:2, but is thought to depend on the local availability of 18:2-containing phospholipid donors for the transacylation reaction [52].

Tafazzin is commonly described as a cardiolipin remodelling enzyme, and this is biochemically correct. Conceptually, however, it better fits the biosynthetic framework described here. Mature CL in most mammalian tissues, with the notable exception of brain, is not broadly diverse but converges on a highly stereotyped pool of 18:2-rich species [52]. Moreover, tafazzin deficiency causes both reduction of mature CL and accumulation of MLCL, indicating that no alternative mitochondrial LPLAT can effectively replace this step [53–58]. In this sense, tafazzin does not merely tune an existing CL pool; it is required to establish the mature CL pool.

This raises the broader question of why mammalian mitochondrial membranes are organised to channel 18:2-containing acyl chains via tafazzin into mature CL. The answer probably relates to the specialised physical requirements of the inner mitochondrial membrane, where CL supports membrane curvature, respiratory chain organisation and protein-lipid interactions. Linoleate-rich CL may provide an appropriate balance between flexibility, packing and resistance to oxidative damage. More unsaturated PUFAs such as arachidonate or DHA might increase fluidity further, but may also render CL excessively susceptible to peroxidation in the reactive oxygen species-rich mitochondrial environment.

### LPCAT2/LPLAT9 and platelet-activating factor

Platelet-activating factor (PAF; 1-*O*-alkyl-2-acetyl-*sn*-glycero-3-phosphocholine) is a potent phospholipid mediator of inflammation. In inflammatory cells, PAF is produced mainly through what is classically termed the ‘remodelling pathway’. Cytosolic phospholipase A2 generates lyso-PAF from alkyl-PC, and lyso-PAF is then acetylated by LPCAT2, also termed LPLAT9 [59]. Despite this name, lyso-PAF acetylation is a committed biosynthetic step: it generates a defined lipid mediator whose *sn*-2 acetyl group is essential for its activity. LPCAT2 can also incorporate arachidonate into PC, giving it a role in both PAF synthesis and phospholipid remodelling. Both activities are acutely enhanced by Ser34 phosphorylation in response to inflammatory stimuli [60,61], and LPCAT2-dependent PAF production has been implicated in neuropathic pain [62]. LPCAT2 thus illustrates how a single LPLAT can contribute to both modes, with the balance set by substrate availability and cellular state.

### CLN8, CLN5 and bis(monoacylglycero)phosphate

Bis(monoacylglycero)phosphate (BMP) is an endolysosomal phospholipid with unique *S,S*-stereochemistry and important roles in lysosomal catabolism [63]. BMP has also emerged as a protective factor in neurodegeneration: its levels rise in multiple neurodegenerative settings, likely as an adaptive response to lysosomal stress, and experimental BMP elevation can improve disease phenotypes in preclinical models [64–68]. Despite this importance, the biosynthetic pathway of BMP has remained poorly defined.

It has long been appreciated that lysosomal BMP is derived from PG, a mitochondrial phospholipid [69,70]. However, whereas PG is typically enriched in SFAs and MUFAs, BMP displays marked tissue-specific acyl compositional variation and is often highly enriched in DHA-containing species, particularly in brain [71]. Thus, substantial acyl chain remodelling occurs during conversion of PG to BMP.

Two acyltransferase enzymes now place this pathway into a clearer biosynthetic framework: CLN8 and CLN5. Both are encoded by genes mutated in Batten disease, a group of neurodegenerative lysosomal storage disorders, and loss of either enzyme causes loss of cellular BMP [7,72–74]. These proteins differ strikingly in localisation and chemistry. CLN8 is an ER membrane protein that functions as an acyl-CoA-dependent acyltransferase, whereas CLN5 is a soluble lysosomal lumenal transacylase [72,74].

Mechanistically, CLN8 catalyses stereospecific acylation of glycerophosphoglycerol (GPG) to form *S,S*-lysophosphatidylglycerol (*S,S*-LPG) [72]. *S,S*-LPG is then delivered to lysosomes by a route that remains to be defined, where CLN5 generates *S,S*-BMP by transferring an acyl chain from one *S,S*-LPG molecule onto the glycerol headgroup of another, releasing *S,S*-GPG as a by-product [72,74]. Consistent with their biosynthetic roles, loss of either CLN8 or CLN5 abolishes cellular BMP production.

This places CLN8 in an interesting conceptual position. Strictly speaking, CLN8 is not a lysophospholipid acyltransferase, because its acceptor, *S,S*-GPG, is fully deacylated. It is, however, a close paralogue of TLCD1, discussed above in the context of classical acyl chain remodelling, and shares the same overall structural fold and catalytic chemistry (Figure 2) [7,72]. Yet in the BMP pathway, this acyltransferase chemistry is embedded within a committed biosynthetic route to a distinct lysosomal lipid. CLN8 and TLCD1 therefore illustrate how closely related enzymes can use similar catalytic mechanisms to produce very different biological outcomes depending on pathway context (Figure 2).

**Figure 2:**
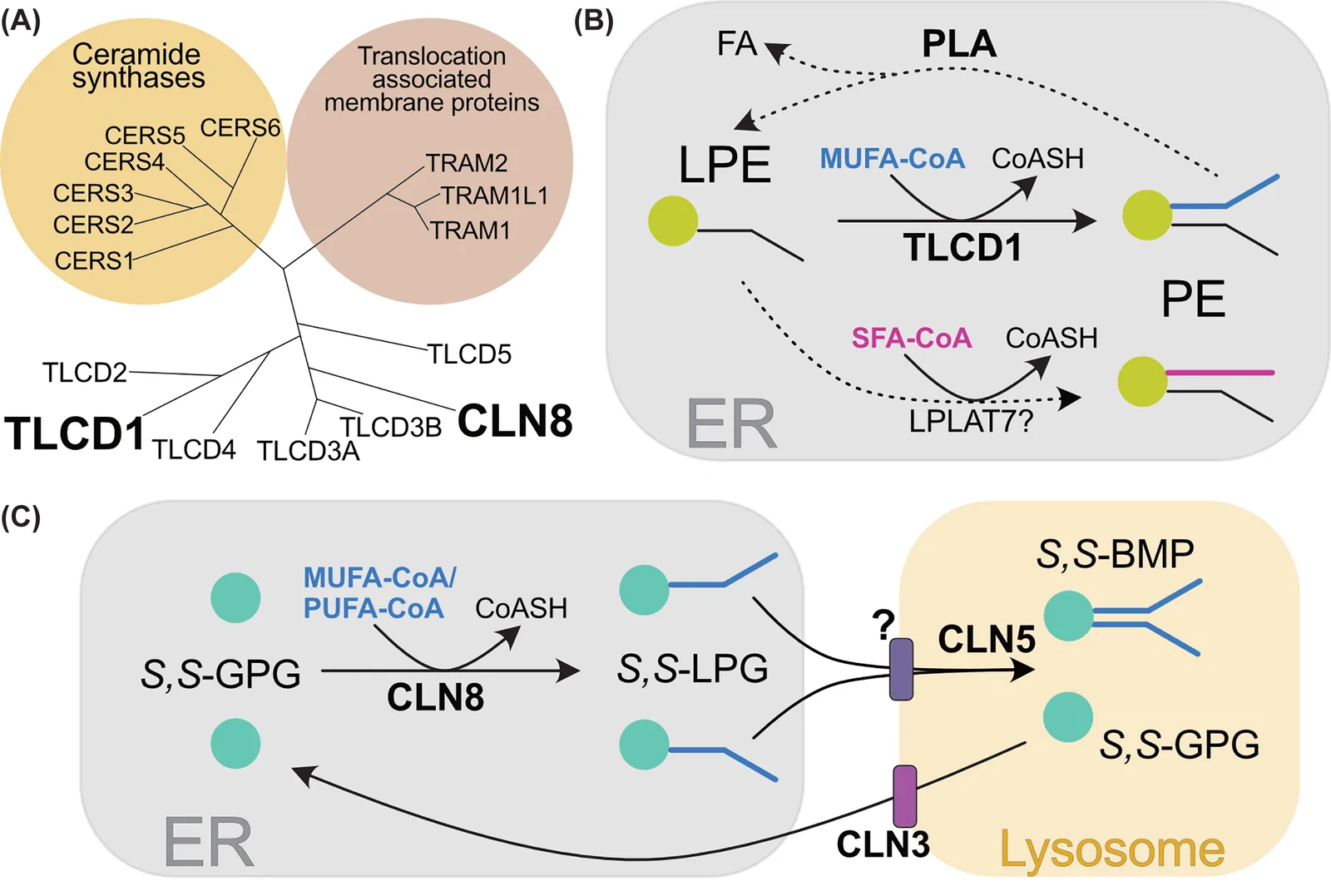
Closely related TRAM–Lag1–CLN8 family enzymes illustrate the distinction between phospholipid remodelling and biosynthesis (**A**) TLCD1 and CLN8 are paralogous members of the TRAM–Lag1–CLN8 family. They share a homologous membrane-embedded catalytic domain, conserved active site residues and an analogous ping-pong acyl transfer mechanism. (**B**) TLCD1 exemplifies phospholipid remodelling. At the endoplasmic reticulum (ER), TLCD1 transfers monounsaturated fatty acyl (MUFA) groups to lysophosphatidylethanolamine (LPE), generating phosphatidylethanolamine (PE) species with monounsaturated fatty acyl chains at the *sn*-1 position. When TLCD1 activity is reduced, total PE abundance is largely maintained because alternative ER-localised LPLATs, such as LPLAT7, can use the same LPE substrate. This preserves total PE abundance but shifts the *sn*-1 acyl chain composition from monounsaturated to saturated fatty acyl (SFA) containing species. (**C**) CLN8 instead exemplifies phospholipid synthesis. CLN8 catalyses the acylation of *S,S*-glycerophosphoglycerol (*S,S*-GPG) at the ER to generate *S,S*-lysophosphatidylglycerol (*S,S*-LPG). *S,S*-LPG is then trafficked to the lysosomal lumen via an unknown route, where CLN5 catalyses transacylation between two *S,S*-LPG molecules to produce *S,S*-bis(monoacylglycero)phosphate (*S,S*-BMP) and *S,S*-GPG. The *S,S*-GPG byproduct is then exported out of the lysosome in a CLN3-dependent manner [75] and returns to the ER, where it can be reused as a CLN8 substrate. Consistent with this dedicated pathway architecture, loss of either CLN8 or CLN5 abolishes cellular BMP synthesis.

At the same time, BMP highlights an important intersection between phospholipid biosynthesis and acyl remodelling. In contrast to classical Lands cycle remodelling, CLN8 defines BMP acyl composition during biosynthesis itself, because the acyl chains it introduces into *S,S*-LPG are retained in the mature BMP molecule [72]. Its preference for 22:6-CoA therefore provides a direct explanation for the DHA enrichment of BMP in brain. This in turn raises two important questions. First, why are DHA-rich BMP species selected, and what do they contribute to lysosomal membrane function? Second, different tissues and cell types show strikingly different BMP species profiles, even though CLN8 is the only known enzyme that determines which acyl chains enter the BMP biosynthetic pathway. Differences in accessible acyl-CoA pools are likely to contribute to BMP acyl diversity, but are unlikely to explain the full extent of this variation. This suggests that additional mechanisms shaping BMP acyl composition remain to be identified, and that these are not readily explained by our current understanding of classical Lands cycle remodelling.

## Conclusions

As lipidomics and mechanistic cell biology continue to advance, phospholipid acyl composition is being examined in an increasingly broad range of physiological and disease contexts. In parallel, more acyltransferases are being identified, their substrate preferences defined, and their loss-of-function phenotypes characterised in cells and organisms. These developments make it timely to revisit the language used to interpret lysophospholipid acylation reactions.

A useful framework is to ask, for each LPLAT under study, whether its loss reduces the final phospholipid product, whether its lysophospholipid substrate accumulates, whether other enzymes can compensate, whether acyl composition is being diversified or instead directed towards a defined lipid endpoint, and whether these answers change with cell type or physiological state. Making this distinction should help sharpen experimental questions and improve interpretation of lipidomic datasets. More broadly, it should help shift the field away from viewing phospholipid acyl chains as secondary decorative features, and towards understanding them as encoded determinants of membrane properties, organelle identity and cellular function.

## Perspectives

- LPLATs are central determinants of phospholipid acyl chain composition, shaping membrane biophysical properties, organelle identity and lipid-dependent cellular processes.
- Lysophospholipid acylation should not always be interpreted as phospholipid remodelling. In some settings, LPLATs adjust the molecular composition of existing phospholipid classes, whereas in others they act as committed biosynthetic enzymes that generate defined lipid products with specific biological functions.
- Future work should define how individual LPLATs are positioned within cellular pathways, how their substrate access and acyl-CoA selectivity are controlled, and how changes in specific phospholipid species contribute to membrane properties, organelle physiology and disease mechanisms.

## Open Access

Open access for this article was enabled by the participation of the University of Cambridge in an all-inclusive Read & Publish agreement with Portland Press and the Biochemical Society under an agreement with JISC.

## Abbreviations

Acyl-CoA: Acyl-coenzyme A
AGPAT: 1-Acylglycerol-3-phosphate O-acyltransferase
ALE2: Yeast lysophosphatidylethanolamine acyltransferase related to TLCD1
BMP: bis(monoacylglycero)phosphate
CERS: Ceramide synthase
CL: Cardiolipin
CLN: Neuronal ceroid lipofuscinosis protein
CoA: Coenzyme A
CoASH: Free coenzyme A
DGAT: Diacylglycerol acyltransferase
DHA: Docosahexaenoic acid
ER: endoplasmic reticulum
FA: Fatty acid
GPG: Glycerophosphoglycerol
LAG1: Longevity assurance gene 1
LCLAT: Lysocardiolipin acyltransferase
LPAAT: Lysophosphatidic acid acyltransferase
LPC: Lysophosphatidylcholine
LPCAT: Lysophosphatidylcholine acyltransferase
LPE: lysophosphatidylethanolamine
LPEAT: Lysophosphatidylethanolamine acyltransferase
LPG: Lysophosphatidylglycerol
LPGAT: Lysophosphatidylglycerol acyltransferase
LPI: Lysophosphatidylinositol
LPLAT: lysophospholipid acyltransferase
LYCAT: Lysocardiolipin acyltransferase
Lyso-PAF: Lysoplatelet-activating factor
MBOAT: Membrane-bound O-acyltransferase
MLCL: Monolysocardiolipin
MUFA: monounsaturated fatty acyl
PAF: Platelet-activating factor
PC: Phosphatidylcholine
PE: phosphatidylethanolamine
PG: Phosphatidylglycerol
PI: Phosphatidylinositol
PLA: Phospholipase A
PUFA: Polyunsaturated fatty acid
SFA: Saturated fatty acid
TAZ: Tafazzin
TLCD: TRAM–LAG1–CLN8 domain-containing
TRAM: Translocating chain-associated membrane protein
